# An intranasal cationic liposomal polysaccharide vaccine elicits humoral immune responses against *Streptococcus pneumoniae*

**DOI:** 10.1038/s42003-024-06806-1

**Published:** 2024-09-17

**Authors:** Peng Wei, Cecilia Romanò, Chengxin Li, Gael Clergeaud, Thomas L. Andresen, Jonas R. Henriksen, Anders E. Hansen, Mads H. Clausen

**Affiliations:** 1 Center for Nanomedicine and Theranostics, Department of Chemistry, Technical University of Denmark, Kongens Lyngby, 2800 Denmark; 2 Department of Chemistry, Technical University of Denmark, Kongens Lyngby, 2800 Denmark; 3 Section for Biotherapeutic Engineering and Drug Targeting, Department of Health Technology, Technical University of Denmark, Kongens Lyngby, 2800 Denmark

**Keywords:** Adjuvants, Bacterial infection

## Abstract

Diseases caused by *S. pneumoniae* are the leading cause of child mortality. As antibiotic resistance of *S. pneumoniae* is rising, vaccination remains the most recommended solution. However, the existing pneumococcal polysaccharides vaccine (Pneumovax^®^ 23) proved only to induce T-independent immunity, and strict cold chain dependence of the protein conjugate vaccine impedes its promotion in developing countries, where infections are most problematic. Affordable and efficient vaccines against pneumococcus are therefore in high demand. Here, we present an intranasal vaccine Lipo^+^CPS12F&*α*GC, containing the capsular polysaccharides of *S. pneumoniae* 12F and the iNKT agonist *α*-galactosylceramide in cationic liposomes. In BALB/cJRj mice, the vaccine effectively activates iNKT cells and promotes B cells maturation, stimulates affinity-matured IgA and IgG production in both the respiratory tract and systemic blood, and displays sufficient protection both in vivo and in vitro. The designed vaccine is a promising, cost-effective solution against pneumococcus, which can be expanded to cover more serotypes and pathogens.

## Abstract (web summary)

Intranasal immunization with capsular pneumococcal polysaccharides 12F and iNKT agonist *α*-galactosylceramide in cationic liposomes protects mice from S. pneumoniae 12F infection.

## Introduction

S*treptococcus pneumoniae*, or pneumococcus, causes both noninvasive (pneumonia, otitis media, sinusitis, and conjunctivitis) and invasive diseases (meningitis, sepsis, and osteomyelitis), which are estimated to kill 1,000,000 children every year^1^. Its capsular polysaccharide is the most virulent component, with over 100 different repeating unit structures, determining its serotype^2^. *S. pneumoniae* has developed resistance against all antibiotics except vancomycin, and in many countries, macrolide antimicrobials, penicillins, and cephalosporins can no longer be assumed effective^3^. In the face of increasing antibiotic resistance, vaccines are becoming a viable alternative against *S. pneumoniae*.

Pneumococcal polysaccharide vaccines (Pneumovax^®^ 23) and conjugate vaccines (Prevnar^®^ 7, 13, 20, & Vaxneuvance™ 15) are the most widely used vaccines. Pneumovax^®^ 23, comprising a mixture of capsular polysaccharides from 23 different serotypes of pneumococcus without adjuvant, has weak immunogenicity (56 % effectiveness^4^) because the classical major histocompatibility complex (MHC) molecule cannot present carbohydrate-based antigens, which is central for inducing T-dependent adaptive immunity. To overcome this restriction, the carbohydrate antigens can be conjugated to a carrier protein (e.g., CRM197, a detoxified variant of diphtheria toxin). Based on this strategy, Prevnar^®^s, Vaxneuvance™ 15, and some preclinical vaccines^5–10^ were developed. In clinical studies, Prevnar 13 displayed improved overall efficacy, but it still has limitations when used in elderly individuals (46 % risk reduction^11^).

Another approach is to link the carbohydrate antigen to an immunogenic lipid, such as *α*-galactosylceramide (*α*GC), which can activate semi-invariant natural killer T (iNKT) cells and further initiate the iNKT-assisted adaptive immune responses. Several semisynthetic vaccines^12–15^ using this approach have shown promising results. However, their fabrication processes contain chemical synthesis, which can increase the cost and restrict availability to vulnerable populations in low-income areas. Notably, all currently licensed pneumococcal vaccines are not stable at room temperature and require to be stored and delivered within a cold chain, which can account for as much as 50% of the total cost^16^. This further presents an unignorable obstacle to their distribution in developing countries, where preventive vaccination is most important.

**Fig. 1:**
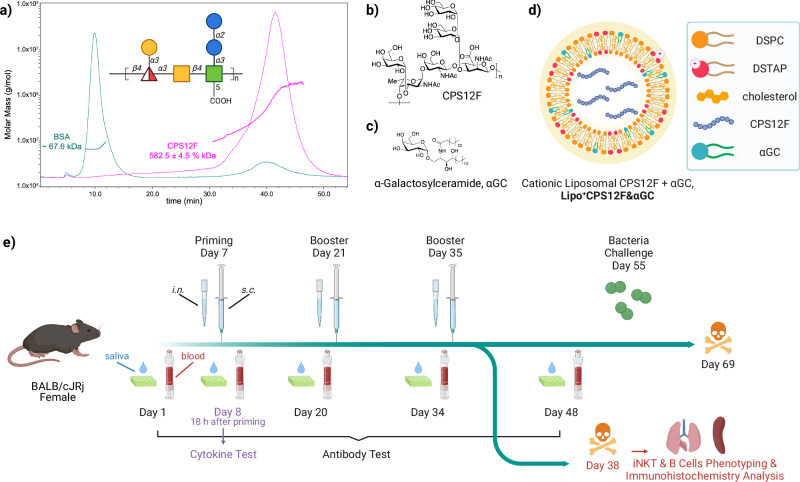
Study design. Molecular weight range **a**) and chemical structure **b**) of the pneumococcus serotype 12F capsular polysaccharide. **c** Chemical structure of the iNKT agonist *α* -galactosylceramide. **d** Formulation of the liposomal vaccine Lipo^+^CPS12F& *α*GC. **e** Scheme of the mouse study. The illustration was created with ChemDraw and BioRender.

This study presents proof of principle for a cost-effective vaccine, Lipo^+^CPS12F&*α*GC (Fig. 1a–d), comprising the capsular polysaccharide of *S. pneumoniae* 12F and the iNKT agonist *α*GC in cationic liposomes. The antigen was extracted from cultured bacteria without additional chemical synthesis, the two components were co-delivered in easily fabricated cationic liposomes, and the working formulation can be stored safely at room temperature for a minimum of 3 weeks. These features help reduce costs and simplify logistics. Moreover, in contrast to licensed Pneumovax^®^ and Prevnar^®^s, which are administered intramuscularly or subcutaneously, our vaccine can be intranasally applied with the help of the cationic liposome formulation. This immunization route is more effective in stimulating the local respiratory mucosal immune systems, which are proximal to infection sites.

Lipo^+^CPS12F&*α*GC successfully initiated the iNKT-mediated B cell maturation and produced antigen-specific high-affinity IgA and IgG antibodies in both the mucosal and systemic immune systems. Importantly, in both in vitro and in vivo studies, the vaccine demonstrated efficient protection potential against the pathogen. Our study used capsular polysaccharides from serotype 12F as the prototype, since this strain can cause invasive pneumococcal diseases and cases broke out in many countries^17–20^. We believe our design holds promise for expansion to more serotypes.

## Results

### Molecular weight characterization of CPS12F

The structure of pneumococcus serotype 12F capsular polysaccharides (CPS12F, Fig. 1b) was determined in 1981^21^. In our study, we obtained the CPS12F from SSI Diagnostica (>97%, 76939), which was purified from the reference strain culture. Its average molecular weight was 582.5  ± 4.5% kDa (Fig. 1a) determined by AF4-MALS-dRI.

### Preparation and characterization of cationic liposomal vaccines

The complete formulation was cationic liposomal CPS12F with *α*GC, abbreviated as Lipo^+^CPS12F&*α*GC (Fig. 1d, Table 1). CPS12F (Fig. 1a, b) was the naturally extracted antigen from *S. pneumoniae* 12F, and *α*GC (Fig. 1c) was co-formulated to induce iNKT-mediated immune responses. In the liposome recipe, DSPC was used as the backbone lipid, cholesterol was added to improve the stability, and DSTAP was included to introduce positive charges on the liposome surface. Group 3, using CPS12F without *α*GC, was included to mimic the approach employed in the Pneumovax^®^ 23. The vaccine was designed to be administered through the *i.n*. route.

**Table 1:** List of vaccine formulations tested in the mouse study

Group	Antigen	Adjuvant	Liposome	Route
1 (placebo)	–	–	cationic	*i.n*.
2	–	*α*GC	cationic	*i.n*.
3	CPS12F	–	cationic	*i.n*.
4	CPS12F	*α*GC	neutral	*i.n*.
5 (preferred)	CPS12F	*α*GC	cationic	*i.n*.
6	CPS12F	*α*GC	cationic	*s.c*.

**Fig. 2:**
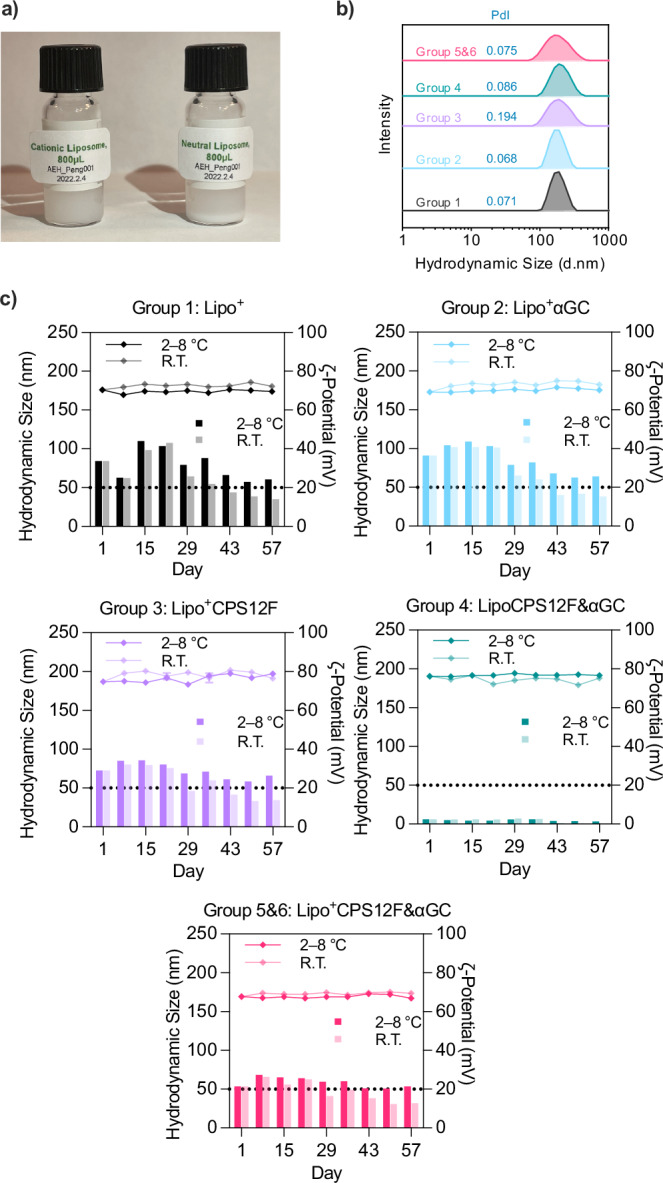
Size distribution, *ζ*-potential, and stability of liposomal vaccines. **a** Photo of cationic and neutral liposomal vaccine solution after static placement. **b** Hydrodynamic size distribution of liposomal vaccines characterized via DLS with a zetasizer. **c** Liposomal vaccines were stored at 2–8 ^∘^C (in a fridge) or 18–24 ^∘^C (at room temperature). The hydrodynamic size (left axis, curves above) and *ζ*-potential (right axis, bars below) were measured weekly for 2 months with a zetasizer.

After static placement (Fig. 2a), the neutral liposomes were deposited to the bottom; in contrast, the cationic liposomes remained in the suspension, presumably because their positive *ζ*-potential prevented aggregation through electrostatic repulsion. However, the precipitation profile did not indicate the neutral liposome was less stable than the cationic liposome. Their homogeneity could be easily restored by vortexing or pipetting.

The stability of the liposomes was further assessed in a zetasizer. Liposomal vaccines of all groups displayed centralized hydrodynamic size distribution ranging from 150 to 200 nm (Fig. 2b) with polydispersity indexes (PdI) below 0.2 (normally, PdI  < 0.3 is considered homogeneous^22^). Figure 2c demonstrated that the hydrodynamic size of all liposomal vaccines did not undergo changes after storage at either room temperature (18–24 ^∘^C) or in the fridge (2–8 ^∘^C). The *ζ*-potential tended to decrease over time under both conditions. However, they remained above 20 mV for at least 2 months in the fridge and 3 weeks at room temperature. The storage length was superior to both the widely applied liposomal vaccine (12 h at room temperature) for COVID-19 and the non-liposomal vaccine Prevnar^®^ 20 (96 h at room temperature) for pneumonia by Pfizer^23,24^. This improvement potentially promotes accessibility for individuals in areas lacking cold chain logistics during the final stages of delivery, and may also lower the cost of the vaccine.

### Lipo^+^CPS12F&*α*GC increased pro-inflammatory cytokines secretion compared to both Lipo^+^ and Lipo^+^CPS12F controls

**Fig. 3:**
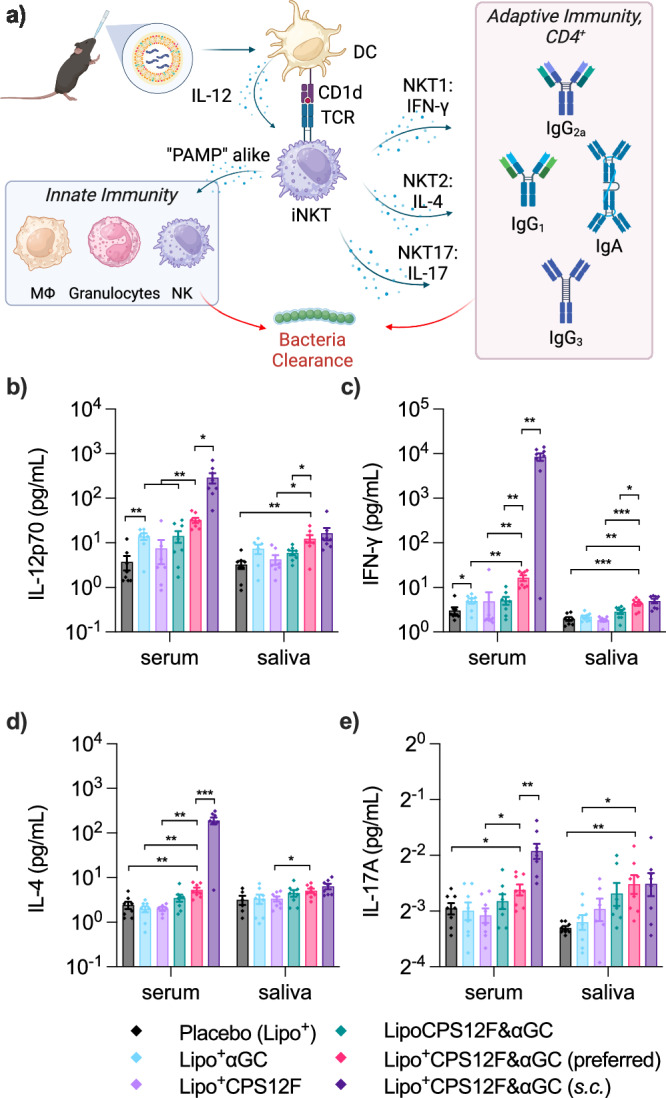
Lipo^+^CPS12F& *α*GC stimulated elevated pro-inflammatory cytokine secretion compared with other formulations. **a** Illustration of cytokines secretion after vaccination, created with BioRender. Concentrations of **b** IL-12p70, **c** IFN-*γ*, **d** IL-4, and **e** IL-17A in serum and saliva. BALB/cJRj mice were immunized with Lipo^+^ (group 1, blank cationic liposomes as placebo), Lipo^+^*α*GC (group 2), or 3 nmol antigen (repeat units of the polysaccharides) in Lipo^+^CPS12F (group 3), LipoCPS12F& *α*GC (group 4), or Lipo^+^CPS12F& *α*GC (group 5 & 6) via intranasal instillation (*i.n*., group 1–5) or subcutaneous injection (*s.c*., group 6). Serum and saliva were collected 18 hours after the priming vaccination, and IL-12p70, IFN-*γ*, IL-4, and IL-17A inside were quantified via cytometric bead array flow cytometry. Data were plotted as mean  ± SEM (*n* = 8). Welch and Brown-Forsythe ANOVA with multiple comparisons tests were done to assess statistical significance. Group 5 was the preferred group. *, **, ***, **** represent *P* < 0.05, *P* < 0.01, *P* < 0.001, *P* < 0.0001.

We first investigated if Lipo^+^CPS12F&*α*GC can invoke cells to secrete pro-inflammatory cytokines, including IL-12, IFN-*γ*, IL-4, and IL-17. As depicted in Fig. 3a, antigen-presenting cells (e.g., dendritic cells) secrete IL-12, which helps iNKT cell activation and proliferation; iNKT cells further secrete IFN-*γ*, IL-4, and IL-17, which separately promote their differentiation into NKT1, NKT2, and NKT17. Combinations of the cytokines will create different cytokine milieus, which will steer the final immune responses^25^.

The strategy to target iNKT is promising in carbohydrate vaccine development. Firstly, iNKTs are “pre-primed” and respond faster than normal T cells; while T_FH_ helps form germinal centers in 10 days, NKT_FH_ only takes 3 days^26^. Secondly, activating iNKTs will result in a universal effect, as its ligand CD1d is more conserved in structure than the conventional MHC molecule^27^. Most importantly, iNKT can both activate innate immunities by the “pathogen-associated molecular patterns (PAMPs) like” functions and differentiate into “T-helper” phenotypes to contribute to adaptive immune responses. The bridging capability between innate and adaptive immunity can result in a synergistic effect and improve bacterial clearance efficacy^28^.

Lipo^+^CPS12F&*α*GC, when delivered intranasally (group 5) or subcutaneously (group 6), both induced significant elevations of IL-12, IFN-*γ*, IL-4, and IL-17 levels (Fig. 3b–e) compared to the placebo group (group 1), with the exception of salivary IL-4 (*P* = 0.06). Compared with the *s.c*. route, the *i.n*. route raised the levels of all four cytokines (Fig. 3b–e) in the saliva but fewer in the serum, which represents the respiratory mucosal (nasal- and inducible bronchus-associated lymphoid tissue, NALT and iBALT) and systemic immune responses separately. It indicated the *i.n*. route is suitable for inducing localized airway mucosal immune responses. The cationic formulation (group 5) showed greater potential in stimulating IL-12 and IFN-*γ* compared to the neutral formulation (group 4, Fig. 3b, c). However, it only displayed trends towards significance in serum IL-4 production (*P* = 0.07) and not significant results in others. The secretion of IL-12 and IFN-*γ* displayed a strong correlation with iNKT agonist *α*GC, as the cationic liposomal *α*GC (group 2) independently induced the production of serum IL-12 and IFN-*γ* (Fig. 3b, c, the salivary results displayed trends but were not significant, *P* = 0.07).

Overall, these results showed that Lipo^+^CPS12F&*α*GC successfully stimulated the secretion of pro-inflammatory cytokines. The liposome surface charge, *α*GC, and immunization route all played a role in this effect. The combination of these cytokines could further activate immune cells and enhance both innate and adaptive immune responses.

### Lipo^+^CPS12F&*α*GC activated iNKTs and induced B cells maturation

We then assessed whether Lipo^+^CPS12F&*α*GC could activate iNKT and B cells in the systemic and mucosal immune systems. Similar to conventional T_FH_ cells, activation of iNKTs (CD69/25^+^^29^) and their differentiation into NKT_FH_ (CXCR5^+^) provided critical and prolonged support^26^ of B cells during its somatic hypermutation into mature plasma cells (CD27^+^CD138^+^), which produce and secrete high-affinity IgA and IgG antibodies.

**Fig. 4:**
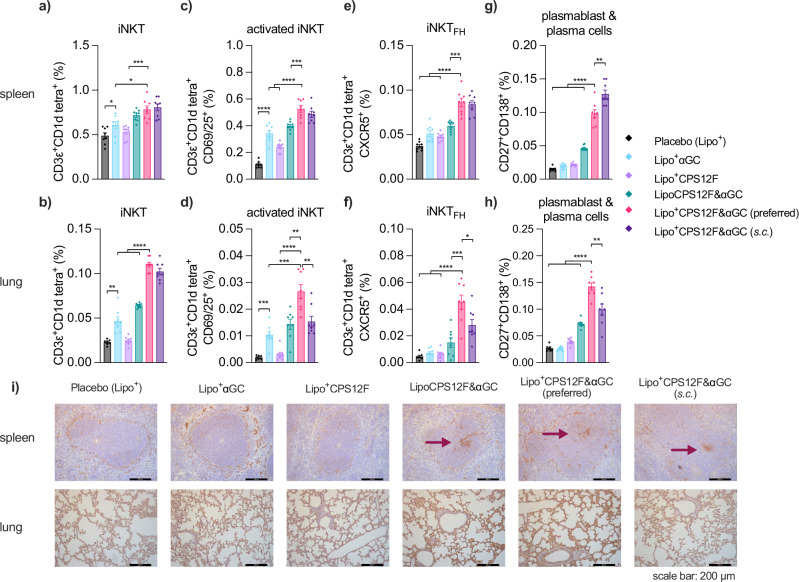
Lipo^+^CPS12F& *α*GC initiated a higher level of iNKT cell activation and B cell maturation compared with other formulations. Percentage of **a** and **b** iNKT cells (CD3*ε*^+^CD1d tetra:*α*GC^+^), **c** and **d** activated iNKT cells (CD3*ε*^+^CD1d tetra:*α*GC^+^CD69/25^+^), **e** and **f** follicular helper iNKT cells (CD3*ε*^+^CD1d tetra:*α*GC^+^CXCR5^+^), and **g** and **h** plasmablast and plasma cells (CD27^+^CD138^+^) to total viable cells in the spleen and lung, separately. **i** Germinal centers (marked by arrows) formed in spleens and morphology of lung tissues after vaccinations. BALB/cJRj mice were immunized with Lipo^+^ (group 1, blank cationic liposomes as placebo), Lipo^+^*α*GC (group 2), or 3 nmol antigen (repeat units of the polysaccharides) in Lipo^+^CPS12F (group 3), LipoCPS12F& *α*GC (group 4), or Lipo^+^CPS12F& *α*GC (group 5 & 6) via intranasal instillation (*i.n*., group 1–5) or subcutaneous injection (*s.c*., group 6) for 3 times at 2 weeks intervals. Spleen and lung were isolated 3 days after the final vaccination and analyzed via multi-color flow cytometry and immunohistochemistry. Data were plotted as mean  ± SEM (*n* = 8). Welch and Brown–Forsythe ANOVA with multiple comparisons tests were done to assess statistical significance. Group 5 was the preferred group. *, **, ***, **** represent *P* < 0.05, *P* < 0.01, *P* < 0.001, *P* < 0.0001.

Compared with mice that received the placebo formulation (group 1), mice vaccinated with Lipo^+^CPS12F&*α*GC (group 5 & 6) exhibited increased levels of iNKTs proliferation (Fig. 4a, b), activation (Fig. 4c, d), differentiation into the follicular helper phenotype (Fig. 4e, f), and B cells maturation into plasmablast and plasma cells (Fig. 4g, h). For iNKTs, in the spleen, the effect between the intranasally (group 5) and subcutaneously (group 6) vaccinated group was comparable since no statistically significant difference was observed; in contrast, in the lung, results showed that *i.n*. vaccination tended to be more effective than *s.c*. vaccination. For B cells, the correlation between the vaccination route and response location was more evident, as the *s.c*. and *i.n*. vaccination generated more plasma cells in the spleen and lung, respectively. Also, results revealed that the cationic liposomal formulation (group 5) displayed a higher efficiency in both iNKT and B cells stimulation than the neutral liposomal formulation (group 4, the spleen iNKT results displayed trends but not significant, *P* = 0.07), which might be due to its help in the attachment to the negatively charged airway mucosal. It is noticeable that the antigen alone group (group 3) elicited small iNKT and B cells responses, which verified that carbohydrate antigen is poorly immunogenic and hardly activates adaptive immunity as the classical MHC molecule can not present it.

We also examined the spleens and lungs of vaccinated mice (Fig. 4i). Germinal centers, where B cells mature into plasma cells, were detected in the spleens of mice from group 4 to 6, proving the effectiveness of the vaccine formulations and that applying them intranasally triggered systemic immune responses similar to the subcutaneous injection. The lung sections displayed an intact morphology, suggesting the intranasal delivery of the vaccine components was safe. Although the flow cytometry result showed the activation of mucosal immunity, we did not find the inducible bronchus-associated lymphoid tissue (iBALT) in the lung sections. It was likely due to the fact that iBALT is a rare, inducible, and temporary structure which only presents during immune responses and disappears afterward^30^.

To conclude, Lipo^+^CPS12F&*α*GC effectively initiated the iNKT expansion and activation, as well as B cell maturation into plasma cells in both the mucosal and systemic immunity. Similar to the results of cytokine analysis, the contributions from liposome surface charge, *α*GC, and immunization route were also observed here.

### Lipo^+^CPS12F&*α*GC induced isotype-switched high-affinity antibodies against CPS12F

We next checked the change of antibody levels after priming-booster vaccinations and their isotype composition 2 weeks after being fully immunized.

**Fig. 5:**
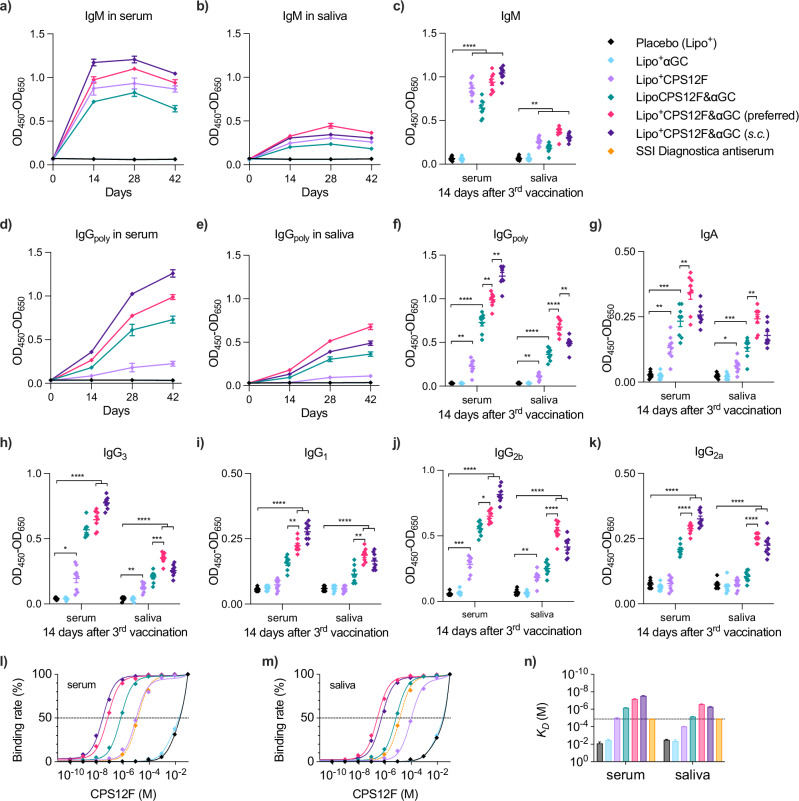
Lipo^+^CPS12F& *α*GC induced superior high-affinity CPS12F-specific antibody production compared with other formulations. Change of CPS12F-specific **a** and **b** IgM and **d** and **e** IgG_poly_ in serum and saliva after immunization. Levels of CPS12F-specific **c** IgM, **f** IgG_poly_, **g** IgA, **h** IgG_3_, **i** IgG_1_, **j** IgG_2b_, **k** IgG_2a_ in serum and saliva 2 weeks after the final vaccination. Binding rate curve for CPS12F against **l** serum antibody and **m** saliva antibody. **n** Dissociation constants (*K*_*D*_) calculated for antibodies in serum and saliva. BALB/cJRj mice were immunized with Lipo^+^ (group 1, blank cationic liposomes as placebo), Lipo^+^*α*GC (group 2), or 3 nmol antigen (repeat units of the polysaccharides) in Lipo^+^CPS12F (group 3), LipoCPS12F& *α*GC (group 4), or Lipo^+^CPS12F& *α*GC (group 5 & 6) via intranasal instillation (*i.n*., group 1–5) or subcutaneous injection (*s.c*., group 6) for 3 times at 2 weeks intervals. Serum and saliva were collected at the start of the study and 2 weeks after each vaccination. Antibodies inside were quantified via indirect ELISA. Their affinities were evaluated via competitive ELISA, in which the binding rate refers to the ratio of [Ab bound with suspension CPS12F]/[Ab bound with bottom CPS12F], and the *K*_*D*_ equals the half-binding concentration of CPS12F. Rabbit antiserum against CPS12F from SSI Diagnostica was used as an external control. Data were plotted as mean  ± SEM (*n* = 8 for **a**–**k**, DF = 29 for **l**–**n**). Curves were fitted using the one-site total binding model. Welch and Brown–Forsythe ANOVA with multiple comparisons tests were done to assess statistical significance. Group 5 was the preferred group. *, **, ***, **** represent *P* < 0.05, *P* < 0.01, *P* < 0.001, *P* < 0.0001.

All antigen-containing formulations (group 3–6) provoked the secretion of IgM (Fig. 5a–c) and IgG_poly_ (Fig. 5d–f), which were observed in both the systemic and mucosal immune systems. Different from IgM, which peaked 2 weeks after the 1^st^ booster vaccination, IgG_poly_ level continued to rise following the priming and booster vaccinations. It validated that IgM is the first responding antibody after stimulation, which can switch into more mature IgG or IgA after B cells go through somatic hypermutation in germinal centers.

IgA is better than IgG in clearing pneumococcus in the respiratory mucosal system. Because IgG mainly functions via complement fixation, opsonization, and their mediated phagocytosis and cytotoxicity (CDC, CDCP, ADCC, and ADCP). During these processes, toxins inside, such as pneumolysin, might be released and cause inflammation. In contrast, IgA is safer as it acts by neutralizing and agglutinating the bacteria, which will finally be removed by ciliary motion^31^.

Vaccination with Lipo^+^CPS12F&*α*GC (group 5 & 6) effectively helped mice produce IgG_poly_ (Fig. 5f) and IgA (Fig. 5g). While the local-distant effect (e.g., *i.n*. tends to bring stronger immune response in MALT) appeared again in the result of IgG_poly_, it is remarkable that mice vaccinated intranasally displayed higher IgA levels in both serum and saliva. Although the differences were not statistically significant, the *p*-value at 0.15 in serum and 0.14 in saliva suggested the trend. Both results proved that the *i.n*. route was more promising since it produced more IgG and IgA in the mucosal system. Similar to the results above, the positive *ζ*-potential also increased the secretion of IgG_poly_ and IgA, further confirming the cationic liposome platform’s capability to deliver mucosal vaccine components.

BALB/c mice have four IgG subclasses (3, 1, 2b, and 2a) determined by the heavy chain constant region gene expression on chromosome 12. Interestingly, although the antigen-only formulation (group 3) stimulated the secretion of IgG_poly_, the subclasses were T-help-limited early-stage IgG_3_ (complement fixation only) and IgG_2b_ (Fc*γ* R mediated function), not later-stage IgG_1_ (amplified power of the Fc*γ* R mediated function) and IgG_2a_ (limited inflammation)^25,32^. In contrast, Lipo^+^CPS12F&*α*GC induced the production of all four IgG subclasses. These results further confirmed the essential role of *α*GC in antibody maturation.

We further measured the affinity of the antibody in collected samples against CPS12F by competitive ELISA (Fig. 5l & m). Lipo^+^CPS12F&*α*GC provoked the production of antibodies with higher affinities (Fig. 5n: *i.n*. vaccination: *K*_*D*_*serum*_ = 66.9 ± 4.7 × 10^−9^*M*, *K*_*D*_*saliva*_ = 259.5 ± 19.4 × 10^−9^*M*; *s.c*. vaccination: *K*_*D*_*serum*_ = 29.3 ± 2.3 × 10^−9^*M*, *K*_*D*_*saliva*_ = 530.9 ± 49.2 × 10^−9^*M*) than the commercial high-affinity rabbit antiserum (*K*_*D*_ = 132.5 ± 6.4 × 10^−7^*M*) from SSI Diagnostica. The results indirectly proved that the designed carbohydrate vaccine successfully activated the adaptive immune responses, in which the B cells went through the somatic hypermutation and finally secreted the mature high-affinity antibodies. Similar to the results in the experiments above, iNKT agonist *α*GC and the cationic liposome both played roles in generating high-affinity antibodies. Furthermore, while *s.c*. injection generated higher affinity antibodies in serum, *i.n*. instillation produced higher affinity antibodies in saliva. This feature indicated the superiority of *i.n*. vaccination in combating respiratory pathogens.

To sum up, Lipo^+^CPS12F&*α*GC efficiently induced the secretion of isotype-switched and affinity-matured CPS12F-specific IgG and IgA both in systemic and mucosal immune systems, which was enhanced by the iNKT agonist *α*GC and cationic liposome design. The results also suggested that *i.n*. vaccination was more capable of eliciting local airway immune responses.

### CPS-specific antibodies efficiently killed *S. pneumoniae* in vitro

**Fig. 6:**
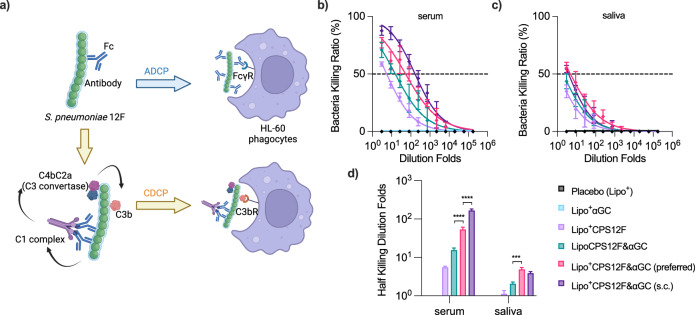
Lipo^+^CPS12F& *α*GC generated antibodies with improved anti-*S. pneumoniae* 12F activity compared with other formulations. **a** Illustration of bacteria-killing mechanisms in the opsonophagocytic killing assay (OPKA), created with BioRender. The ratio of bacteria killed by serum **b** and saliva **c** antibodies mediated opsonization and phagocytosis at different dilution folds. **d** Half-killing dilution folds of the antibodies in serum and saliva samples. BALB/cJRj mice were immunized with Lipo^+^ (group 1, blank cationic liposomes as placebo), Lipo^+^*α*GC (group 2), or 3 nmol antigen (repeat units of the polysaccharides) in Lipo^+^CPS12F (group 3), LipoCPS12F& *α*GC (group 4), or Lipo^+^CPS12F& *α*GC (group 5 & 6) via intranasal instillation (*i.n*., group 1–5) or subcutaneous injection (*s.c*., group 6) for 3 times at 2 weeks intervals. Serum and saliva collected 2 weeks after the final vaccination were pooled-assessed via OPKA (*n* = 8). Data were fitted in the nonlinear dose-response model. Half-killing dilution folds were interpolated and plotted as mean  ± SEM (DF ≥ 38). Welch and Brown-Forsythe ANOVA with multiple comparisons tests were done to assess statistical significance. Group 5 was the preferred group. *, **, ***, **** represent *P* < 0.05, *P* < 0.01, *P* < 0.001, *P* < 0.0001.

We then examined the protective efficacy of the generated antibodies against *S. pneumoniae* in vitro (Fig. 6a & Supplementary Fig. S2).

The antibody is believed to help bacteria clearance by attaching to the pneumococcus surface and mediating phagocytosis by Fc and Fc*γ*R recognition (antibody-dependent cellular phagocytosis, ADCP) or contributing to the formation of complement C3b (classical pathway) and mediating phagocytosis by C3b and C3bR recognition (complement-dependent cellular phagocytosis, CDCP). Their efficiencies were quantified as their half-killing dilution folds (Fig. 6b–d).

The serum and saliva induced by Lipo^+^CPS12F&*α*GC (group 5 & 6) both displayed elevated anti-*S. pneumoniae* 12F activity compared with the *α*GC-lacking (group 3) and neutral liposomal (group 4) formulation, suggesting the significance of iNKT agonist *α*GC and the cationic liposome in improving the immune responses. It is remarkable that, with the same formulation of Lipo^+^CPS12F&*α*GC, while the *s.c*. route (group 6) produced more effective serum, the *i.n*. administration (group 5) tended to produce more effective saliva. However, the difference in saliva tests was not statistically significant.

The result verified the anti-*S. pneumoniae* efficiency of the serum and saliva collected from mice immunized with Lipo^+^CPS12F&*α*GC. The *i.n*. route was more promising, as it mainly stimulated the respiratory mucosal immune system, which was proximal to the infection and disease site.

### Lipo^+^CPS12F&*α*GC vaccination protected mice from *S. pneumoniae* challenge

We finally evaluated the in vivo protective potential of Lipo^+^CPS12F&*α*GC. BALB/cJRj mice were fully vaccinated with different vaccines intranasally (group 1–5) or subcutaneously (group 6) and then challenged with *S. pneumoniae* 12F via *i.n*. instillation.

**Fig. 7:**
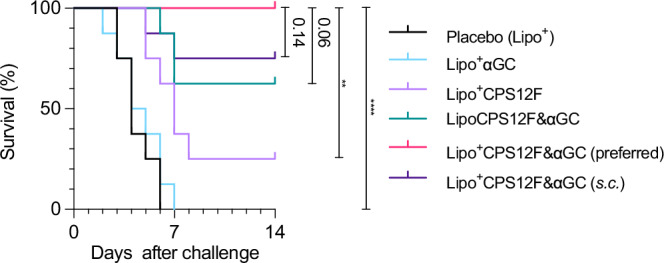
Lipo^+^CPS12F& *α*GC vaccination protected mice from *S. pneumoniae* 12F challenge. Survival curves of vaccinated mice after bacteria challenge. BALB/cJRj mice were immunized with Lipo^+^ (group 1, blank cationic liposomes as placebo), Lipo^+^*α*GC (group 2), or 3 nmol antigen (repeat units of the polysaccharides) in Lipo^+^CPS12F (group 3), LipoCPS12F& *α*GC (group 4), or Lipo^+^CPS12F& *α*GC (group 5 & 6) via intranasal instillation (*i.n*., group 1–5) or subcutaneous injection (*s.c*., group 6) for 3 times at 2 weeks intervals. Three weeks after the final vaccination, mice (*n* = 8) were challenged with 1 × 10^6^ CFU *S. pneumoniae* 12F via *i.n*. instillation and carefully monitored for the next 14 days. Results were plotted as survival curves. Gehan–Breslow–Wilcoxon nonparametric tests were done to assess statistical significance. Group 5 was the preferred group. *, **, ***, **** represent *P* < 0.05, *P* < 0.01, *P* < 0.001, *P* < 0.0001.

In Fig. 7, it is notable that mice intranasally vaccinated with Lipo^+^CPS12F&*α*GC (group 5) all survived the 2 weeks after infection; on the contrary, mice vaccinated with blank cationic liposome (group 1) or cationic liposomal *α*GC (group 2) all reached the humane endpoint within the first week. It demonstrated the significant (*P* < 0.0001) protection rendered by Lipo^+^CPS12F&*α*GC when applied intranasally. Meanwhile, only 2 of 8 (25 %) mice survived after receiving the formulation without *α*GC (group 3), and 5 of 8 (62.5 %) mice survived after receiving the neutral surface charge formulation (group 4), validating the important roles of the iNKT agonist *α*GC and nasal mucosa attachment facilitating positive *ζ*-potential.

Notably, 6 of 8 (75 %) mice survived after receiving the whole formulation via *s.c*. injection (group 6); compared with group 5, although the difference was not statistically significant, the P value of 0.14 indicated the route of vaccination played a role in the final protection strength. *S. pneumoniae* 12F mainly colonizes and causes disease in the respiratory system. Applying the vaccines intranasally can activate the NALT and iBALT, which is proximal to the infection site and more robust than the remote immune responses elicited by systemic *s.c*. vaccination.

The presence of iNKT cells is crucial for a robust immune response after vaccination, as they play a dual role in pathogen defense. On one hand, they directly engage in the destruction of pathogens. On the other, they are pivotal in enhancing the adaptive immune system’s ability to produce specific antibodies. This dual functionality underscores the poorer protection efficacies observed in the group lacking the iNKT agonist.

These results confirmed that Lipo^+^CPS12F&*α*GC, when immunized intranasally, can efficiently protect mice from *S. pneumoniae* 12F challenge.

## Discussion

The effort to develop pneumococcal vaccines has continued for over 100 years, which weakened after the introduction of penicillin in the 1940s, but resurged as the problem of antibiotic resistance increased. Capsular polysaccharide is the most virulent component of *S. pneumoniae*; hence it is the most direct and promising target for vaccine development. However, developing carbohydrate-based vaccines is challenging as carbohydrate antigens cannot be presented by classical MHC molecules and were proven to have weak immunogenicity.

To break this limitation, we utilized the iNKT agonist *α*GC and co-delivered it with the capsular polysaccharide antigen in cationic liposomes. Compared with the carrier protein conjugation strategy, which activates normal T cells, the designed vaccine in this paper targeting iNKTs holds several advantages. First, the iNKT-mediated adaptive immune responses are universal among individuals. The classical polymorphic MHC (human leukocyte antigen, HLA in humans) has many alleles, and their frequency distributions vary significantly among different populations^33^. In contrast, the ligand of TCRs on iNKTs, the CD1d (CD1a-e in humans) molecule, is highly structurally conserved, which helps bring a more predictable and consistent immunization efficacy^27^. Second, iNKTs respond faster after stimulation. Upon activation, iNKTs immediately proliferate and differentiate into functional phenotypes within hours, while it takes days for conventional T cells^34^. Moreover, iNKT_FH_ stimulated the germinal center formation by day 3, which generally takes 10 days for conventional T_FH_ cells^26^. Third, the vaccine has attractive costs, making it more accessible to at-risk populations in low-income areas. The capsular polysaccharide is extracted from the bacteria culture instead of being produced by chemical synthesis. The liposome is fabricated by simple extrusion. The whole formulation can be stored safely at room temperature for at least 3 weeks, as a result, relieving the cold chain problem in developing countries during terminal transportation.

Prior studies using CPS4^14^ and CPS14^12,13^ were administered through parenteral ways, such as subcutaneous and intramuscular injections. In comparison, our vaccine was engineered for intranasal delivery. The intranasal route offers a promising alternative as a more effective vaccination strategy for combating mucosa pathogens, which enhances patient compliance and accessibility.

This was an exploratory study aimed at validating our vaccine approach. In order to balance the scope of the investigation with ethical considerations related to animal welfare, we made some choices to reduce the number of mice used. For instance, we focused on serotype 12F rather than a wider range of arrays and only tested the vaccine on adult inbred BALB/cJRj mice instead of neonatal, aged, or wild-type mice. We also delivered the vaccine intranasally by instillation instead of aerosolization and collected saliva samples instead of bronchoalveolar lavage fluids for lower airway immunity evaluation. With the existing verified efficacy, we plan to include more serotypes of antigens using our vaccine approach with additional experimental evaluation in the future.

In conclusion, Lipo^+^CPS12F&*α*GC, when immunized intranasally, initiated iNKT-helped B cells maturation and elicited the production of antigen-specific high-affinity isotype-switched antibodies both in mucosal and systemic immune systems, which were confirmed to be capable of clearing *S. pneumoniae* in vitro and protecting mice from pathogen challenge in vivo. Our vaccine approach is promising to be expanded to more pneumococcal serotypes and other respiratory pathogens.

## Methods

### Capsular polysaccharides characterization

The molecular weight of the capsular polysaccharides 12F (SSI Diagnostica 76939) was characterized by asymmetric flow field-flow fractionation (AF4) coupled with multi-angle light scattering (MALS) and differential refractive index detectors (dRI).

Briefly, phosphate buffer (38 mM Na_2_HPO_4_⋅2H_2_O and 12 mM NaH_2_PO_4_⋅2H_2_O, Sigma-Aldrich^®^ 71643 & 71505) was freshly prepared, filtered through 0.1 μm PVDF membranes (Durapore^®^ VVLP04700), and used as the flush solution. Bovine serum albumin (BSA, Mw 66.5 kDa, Sigma-Aldrich^®^ 05470) was used as the standard control. 100 μL of each sample (dissolved in the flush buffer at 2 mg/mL, filtered through a 0.2 μm PVDF syringe filter, Thermo ScientificTM 4-SF-02(PV)) was injected into the system with a pump (Agilent 1260 Infinity II), separated in the AF4 channel (350 μm spacer, PES 10 kDa MWCO, 17.5 cm long channel, Wyatt 1899), and measured by a differential refractive index detector (Optilab T-rEX from Wyatt) and a MALS detector (TREOS II from Wyatt).

Results were analyzed using the manufacturer’s software ASTRA v7.3, in which the refractive index increments for BSA and CPS12F were set as 0.185 mL/g and 0.150 mL/g, according to the published studies^35^.

### Liposomal vaccines preparation

The liposomal vaccines were prepared using the extrusion method. The protocols were modified from the manufacturer’s instructions^36,37^.

Briefly, 53.5% of DSPC (Avanti Polar Lipids^®^ 850365P), 15 % of DSTAP (Avanti Polar Lipids^®^ 890880P), 30% of cholesterol (Sigma-Aldrich^®^ C8667), and 1.5% of *α*GC (Biosynth^®^ MG15978) at molar ratio were weighed, dissolved, mixed, and stirred in *t*-BuOH/H_2_O (9/1, v/v) mixture at 70 ^∘^C for 30 min, frozen with liquid nitrogen, and lyophilized overnight on a freeze dryer (LaboGene™ ScanVac CoolSafe) to remove the solvent. On the next day, the lyophilized lipid membrane was rehydrated in HEPES saline solution (10 mM HEPES, 150 mM NaCl, pH adjusted to 7.4, and filtered through 0.22 μm membrane), mixed with 1.5% hydrophilic CPS12F at the molar ratio, diluted to a lipid concentration of 40 mM, stirred at 70 ^∘^C (above the phase transition temperatures^38,39^, T_c, DSPC_ = 55 ^∘^C, T_c, DSTAP_ = 62.9 ^∘^C) for 1 hour, and finally extruded through a 0.2 μm hydrophilic membrane (Nuclepore™ 10417004) 21 times in an extruder (Avanti Polar Lipids) at 70 ^∘^C.

### Components quantification

The retained DSPC and DSTAP in liposomal vaccines were measured by determining the phosphorus concentration through the malachite green phosphate assay (Sigma-Aldrich^®^ MAK307), assuming the retaining ratios of the two compounds were the same. The retained cholesterol was measured by coupled enzyme reactions and a fluorometric assay (Sigma-Aldrich^®^ CS0005). The retained *α*GC was quantified by RP-UPLC-MS/MS (reverse-phase ultra-performance liquid chromatography-tandem mass spectrometry, SHIMADZU Nexera-i LC-2040C & LCMS-8045) via multiple reaction monitoring (MRM) using a 2.6 μm C8 column (Kinetex^®^ 00F-4497-AN). The retained CPS12F was determined by the phenol sulfuric acid method^40,41^.

### Size distribution, *ζ*-potential, and stability evaluation

Hydrodynamic size distribution was assessed by dynamic light scattering (DLS) in a zetasizer (Malvern NanoZS) with HEPES saline solution (same as section 2.1). Surface charge was indirectly measured as *ζ*-potential in a zetasizer with HEPES-glucose solution (10 mM HEPES, 5% (w/v) glucose, 1 mM CaCl_2_, pH adjusted to 7.4, and filtered through 0.22 μm membrane). The samples were separately stored at room temperature (18–24 ^∘^C) or in the fridge (2–8 ^∘^C) and repeatedly characterized by the zetasizer for stability evaluation.

### Mouse strain, vaccination route, and sampling method

We have complied with all relevant ethical regulations for animal use. All animal experiments in this study were approved by the Danish Animal Experiments Inspectorate (2020-15-0201-00482-C1 and 2022-15-0201-01249) and complied with Directive 2010/63/EU. 6–8 weeks old BALB/cJRj female mice (JANVIER LABS^®^), after 1 week of acclimation, were randomized into treatment groups. The vaccinations were carried out via subcutaneous (*s.c*.) injections at 50 μL or intranasal (*i.n*.) installations^42^ at 25 μL for each nostril, to provide enough exposure to NALT and iBALT^43^. At the end of the study, all remaining mice were euthanized by cervical dislocation.

For cytokine and antibody quantification, at defined time points (Fig. 1e), blood samples were collected (sublingual bleeding) and processed into the serum; saliva samples were collected with sampling sponges (Nasco^®^ B01245WA) after pilocarpine injection (Medchem Express HY-B0726, *i.p*., 0.36 μg/g of body weight)^44^. Samples were stored at 2–8 ^∘^C for the short term (≤7 days) and −80 ^∘^C for the long term (>7 days).

For organ examination and cell phenotyping, 3 days after the final vaccination, half of the mice were sacrificed, and their lung and spleen were cryopreserved with OCT (CellPath KMA-0100-00A) for immunohistochemistry analysis or prepared into single-cell suspensions for multi-color flow cytometry analysis. Another half of the mice were challenged with bacteria 1 week after the final serum and saliva sampling to evaluate the efficacy of protection in vivo.

### Cytokine quantification

Cytokines (IL-12, IL-4, IFN-*γ*, and IL-17) were quantified with BD™ cytometric bead array (CBA) assay kits (BD Biosciences 562264, 562272, 562233, 562261) via flow cytometry according to the manufacturer’s protocol^45^.

Briefly, serum and saliva samples were collected 18 hours after the priming vaccination, diluted at 5 folds for serum and 10 folds for saliva, captured by antibodies conjugated with beads with specific fluorescent profiles by incubating for 2 h, further loaded with PE-labeled antibodies for concentration evaluation by incubating for 2 h. Samples were prepared in 96-well round-bottom plates (Corning^®^ 3365). Unbounded components were washed away by centrifugation (400 × *g*, 5 min) and discarding the supernatant. Then, samples and standards were acquired on a flow cytometer (BD^©^ LSRFortessa™) through FSC-A, FSC-W, SSC-A, SSC-W, PE-A, APC-A, and APC-Cy7-A channels, and their median fluorescence intensities of PE channel (MFI_PE_) were calculated in FlowJo 10. Finally, the concentrations of the cytokines were interpolated on the fitted standard curves.

### Cell phenotyping

iNKT cells and B cells in the spleen and lung were phenotyped by multi-color flow cytometry.

Briefly, dissected spleens and lungs were prepared into single-cell suspensions by smashing through 70 μm cell strainers (Falcon^®^ 352350). Red blood cells were removed by treating with lysing buffer (Biosciences™ 555899). Then, cells (1 × 10^6^ cells at 50 μL for each test) were treated with Fc block (BD Biosciences™ 553141) to reduce unspecific binding, incubated with fixable viability stain 520 (BD Biosciences™ 564407), and stained with pre-titrated mouse PE-conjugated *α*GC-loaded mCD1d tetramer (Tetramer Shop MCD1d-001), hamster/rat anti-mouse CD3*ε*-BV605, CD69-APC, CD25-BV480, CD45R-APC-Cy7, CD27-BB700, CD138-BV421, and CXCR5-BV711 (BD Biosciences™ 563004, 560689, 566202, 561102, 742135, 562610, & BioLegend^®^ 145529). Samples were prepared in 96-well round-bottom plates (Corning^®^ 3365). Unbounded components were washed away by centrifugation (400 × *g*, 5 min) and discarding the supernatant. After that, samples and controls were acquired on a flow cytometer (BD^©^ LSRFortessa™). Finally, in FlowJo 10, the compensation matrix was determined with single-stained and unstained bead controls (Invitrogen™ 01-333-41), gating strategies (Supplementary Fig. S1) were developed with published OMIP^46^ and FMO controls, and the ratios of the cell populations of interest were calculated.

### Organ examination

Spleens and lungs were examined by immunohistochemistry.

Briefly, cryopreserved spleens and lungs were fixed with 4% (w/v) formaldehyde (Histolab^®^ 02178), dehydrated with a series of ethanol solutions of increasing concentration and Histolab Clear solution (Histolab^®^ 14250) in a tissue processor (Epredia™ Excelsior AS), embedded in paraffin wax (Histology Wax 0587), cut into 3 *μ*m sections with a rotary microtome (Shandon Finesse AS325), and mounted on adhesion slides (Epreida™ J1810AMNZ). Then, the sections were deparaffinized with xylene (Sigma-Aldrich^®^ 534056) and a series of ethanol solutions of decreasing concentration and water rinse, boiled in sodium citrate buffer (10 mM citrate, pH adjusted to 6.0) for 20 min for epitope retrieval, treated with streptavidin and biotin blocking reagents (VectorLabs SP-2002) to block endogenous biotin, treated with 2% (w/v) BSA (Invitrogen™ DS98200) in 0.3% (v/v) Triton X-100 (Sigma-Aldrich^®^ X100) to reduce unspecific binding. After that, the sections were sequentially incubated with the biotinylated peanut agglutinin (PNA, VectorLabs B-1075-5) for germinal center detection, 3% (w/v) hydrogen peroxide (Millipore^®^ 88597) to quench endogenous peroxidase activity, streptavidin-HRP conjugate (Agilent Dako P0397), and developed color with DAB (abcam ab64238). To better visualize the tissue morphology, the slides were counterstained with Mayer’s hematoxylin (lab-made) for 10 s. Unbounded components were washed away with Tris-buffered saline + 0.025 % (w/v) Tween 20 (lab-made) between each step. Afterward, the stained sections were again dehydrated with a series of ethanol solutions of increasing concentration, air dried, and preserved with the mounting medium (Histolab^®^ 00801) and coverslips (Hounisen 2422.2560). Finally, the slides were observed and photoed under a pathology microscope (Leica DMRB and MC170 HD).

### Antibody quantification

CPS12F-specific antibodies in the samples were measured by indirect ELISA.

Briefly, 384-well plates (Nunc™ MaxiSorp™ 464718) were coated with 100 ng CPS12F (SSI Diagnostica 76939) per well, blocked with BSA (Invitrogen™ DS98200); then, diluted testing samples, HRP-conjugated goat anti-mouse IgM (abcam ab97230), IgA (abcam ab97235), IgG_poly_ (abcam ab6789), IgG_3_ (abcam ab97260), IgG_1_ (abcam ab97240), IgG_2b_ (abcam ab97250), IgG_2a_ (abcam ab97255), and TMB chromogen solution (Invitrogen™ SB02) were sequentially added and incubated in the plate. Unbounded components in the well were washed away with PBS +0.05% (w/v) Tween 20 buffer (PBST, Thermo Scientific™ Pierce™ 28352) between each step. After that, 0.16 M sulfuric acid (Thermo Scientific™ SS04) was added to stop the reaction. Finally, OD_450_ in each well was acquired in a microplate reader (TECAN Spark^®^ Cyto) referenced by OD_650_.

### Antibody affinity measurement

The affinities of antibodies in the samples to CPS12F were determined by competitive ELISA, in which the binding kinetics *K*_*d*_ equals the half-binding concentration of CPS12F^47^.

Briefly, diluted testing samples were incubated with serially diluted CPS12F (SSI Diagnostica 76939) and transferred to 384-well plates (Nunc™ MaxiSorp™ 464718, coated with 2 ng CPS12F per well and blocked with BSA, Invitrogen™ DS98200, in advance); then, HRP-conjugated goat anti-mouse IgG_poly_ (abcam ab6789) and TMB chromogen solution (Invitrogen™ SB02) were sequentially added and incubated in the plate. Unbounded components in the well were washed away with PBS +0.05% (w/v) Tween 20 buffer (PBST, Thermo Scientific™ Pierce™ 28352) between each step. After that, 0.16 M sulfuric acid (Thermo Scientific™ SS04) was added to stop the reaction, and OD_450_ in each well was acquired in a microplate reader (TECAN Spark^®^ Cyto) referenced by OD_650_. Finally, the half-binding concentration was interpolated on the fitted sigmoidal & 4 parameters logistical curves.

### Protection efficacy evaluation in vitro

The vaccine’s protective efficacy was evaluated in vitro via the opsonophagocytic killing assay (OPKA). The protocol was modified from the published protocol^48^.

BALB/cJRj mice were immunized with vaccines 3 times at 2 weeks intervals; 2 weeks after the final vaccination, serum and saliva samples were collected and cryopreserved at −80 ^∘^C until use. *S. pneumoniae* 12F (SSI Diagnostica 82201) bacteria was cultured in THY broth (Todd-Hewitt broth (OXOID™ CM0189B) with 0.5% (w/v) yeast extract (BioReagents™ BP1422)) with 5% (v/v) CO_2_ at 37 ^∘^C until its OD_600_ reaches 0.8; then, it was added with 15% (v/v) glycerol (Invitrogen™ 15514011, cryoprotectant), aliquoted at 0.5 mL, cryopreserved at −80 ^∘^C, and quantified by serial dilution culture on THYA plates (THY broth with 1.5% (w/v) agar (BD BACTO™ 214010)) and CFU counting. HL-60 cells (ATCC^®^ CCL-240™) were differentiated into phagocytes with 0.8% (v/v) of DMF (Thermo Scientific™ 210585000) added into the medium for 3-5 days culture. On the day of assay, cryopreserved bacteria were thawed, diluted to 5 ×10^4^ CFU/mL with freshly prepared opsonization buffer (5% (v/v) heat-inactivated FBS (Gibco™ A3160802) and 0.1% (w/v) gelatin (Thermo Scientific™ 410875000) in HBSS (Gibco™ 24020091)), and aliquoted at 10 μL (500 CFU) into 96-well conical-bottom plates. Serum and saliva samples were treated at 56 ^∘^C for 30 min to deactivate endogenous complement, serially diluted by 3 folds at 30 μL (20 μL left), and mixed with bacteria suspension in the plates for 30 min of incubation at room temperature. Then, differentiated HL-60 cells were resuspended in HBSS at 1 ×10^7^ cells/mL, mixed with baby rabbit complement (MP Biomedicals 08642961) at 4/1 (v/v), and aliquoted at 50 μL into the plates for 45 min of coculture (cells/bacteria = 800/1) with 5% (v/v) CO_2_ at 37 ^∘^C. After that, 10 μL of each mixture was spotted on the THYA plates, overlayed with THYA supplemented with T.T.C. (OXOID™ SR0148A), cultured at 37 ^∘^C for 18 h; then, red colonies formed by unphagocytosed bacteria were counted with software NICE^49^. Finally, the half-killing dilution folds were interpolated on the fitted sigmoidal & 4 parameters logistical curves.

### Protection efficacy evaluation in vivo

The vaccine’s protective efficacy was evaluated in vivo through the bacteria challenge study.

Briefly, mice were immunized with vaccines 3 times at 2 weeks intervals; 3 weeks after the final vaccination, each mouse was challenged with 1 ×10^6^ CFU of *S. pneumoniae* 12F via *i.n*. instillation; then, mice were closely monitored for the following 2 weeks. To secure the welfare of the experimental mice, the below criteria were set as the humane endpoints: weight loss  >20%; labored respiration with increased respiratory rate, effort, or strong abdominal breathing; rough hair coat, unkempt appearance, hunched posture; lethargy or persistent recumbency; loss of righting reflex or failure to maintain equilibrium. Finally, the survival curves were drawn accordingly.

### Statistics and reproducibility

At the start of the study, independent animal replicates (*n* = 8) were randomized and weight balanced to ensure reproducibility. The data were analyzed using GraphPad Prism 9. Welch and Brown-Forsythe ANOVA with multiple comparisons tests were done to assess statistical significance in Figs. 1–6. Gehan–Breslow–Wilcoxon nonparametric tests were done to assess statistical significance in Fig. 7. *, **, ***, **** represent *P* < 0.05, *P* < 0.01, *P* < 0.001, *P* < 0.0001.

### Reporting summary

Further information on research design is available in the Nature Portfolio Reporting Summary linked to this article.

## Supplementary information

Supplementary Information

Description of Additional Supplementary Materials

Supplementary Data 1

Reporting Summary

## Data Availability

Numerical source data for all figures in the manuscripts can be found in Supplementary Data 1. All other data is available upon reasonable request.
