# Supplementary material for: An intranasal cationic liposomal polysaccharide vaccine elicits humoral immune responses against Streptococcus pneumoniae
Source: Commun Biol. 2024 Sep 17;7:1158. doi: 10.1038/s42003-024-06806-1 (PMC11405767; doi:10.1038/s42003-024-06806-1)
Supplement: Supplementary file 1 — Supplementary Information [file 42003_2024_6806_MOESM1_ESM.pdf]

# An Intranasal Cationic Liposomal Polysaccharide Vaccine Elicits Humoral Immune Responses against *Streptococcus pneumoniae*

**Supplementary Information**

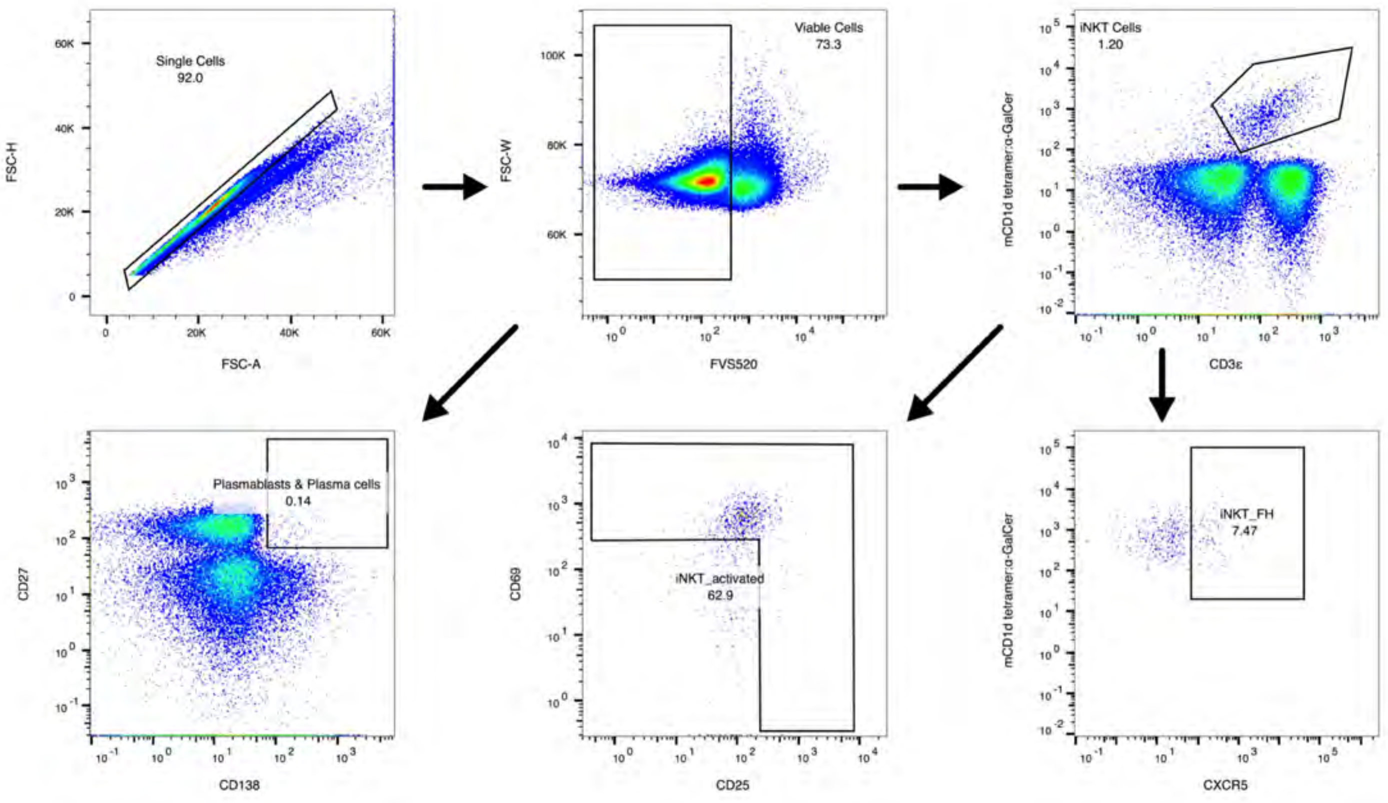


Figure S1: **Gating strategy for the cell phenotyping experiment**

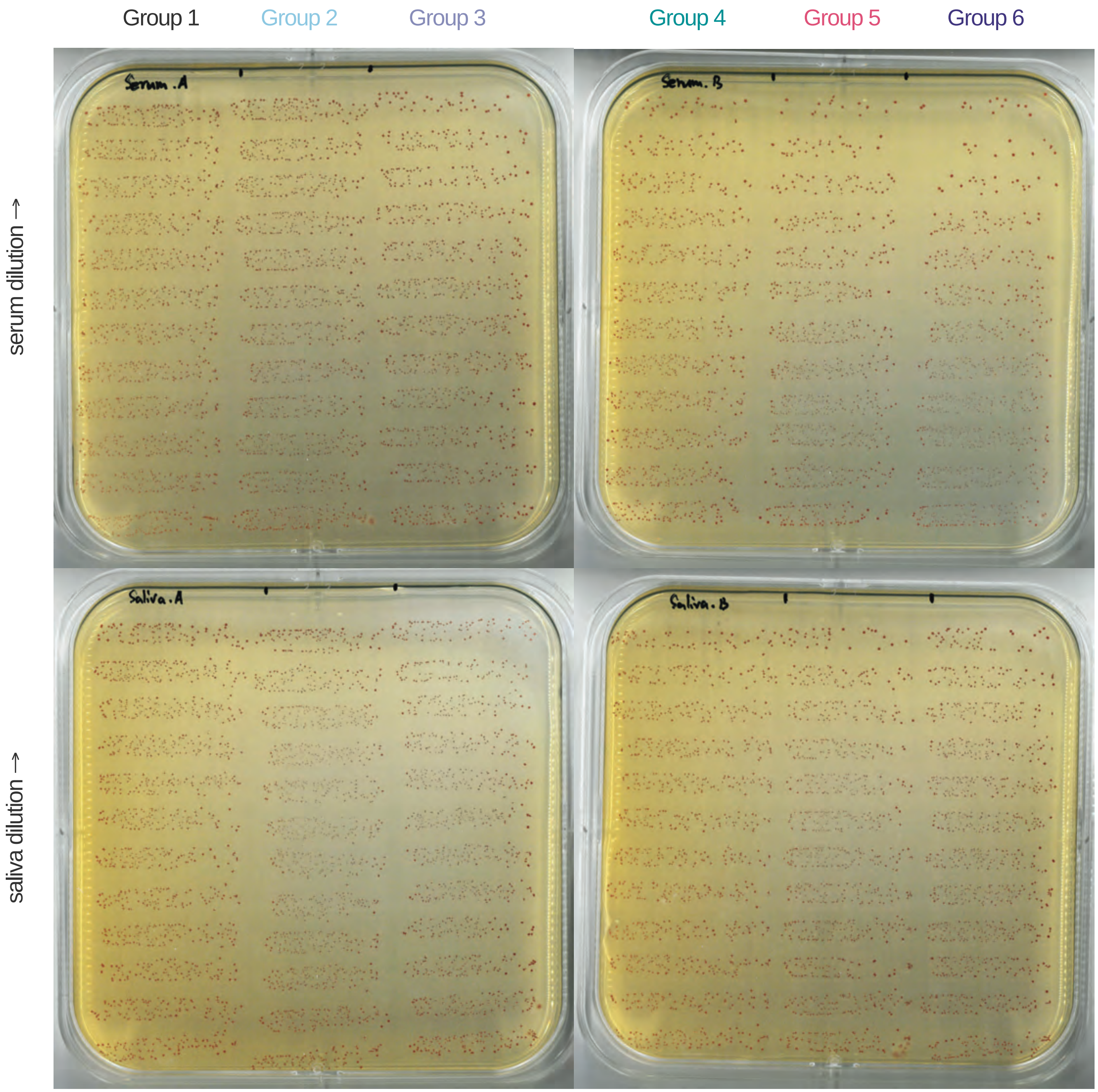


Figure S2: **Colony dots formed by survived *S. pneumoniae* 12F after opsonophagocytosis.** BALB/cJRj mice were immunized with Lipo$^{+}$ (group 1, blank cationic liposomes as placebo), Lipo$^{+}$αGC (group 2), or 3 nmol antigen (repeat units of the polysaccharides) in Lipo$^{+}$CPS12F (group 3), LipoCPS12F&αGC (group 4), or Lipo$^{+}$CPS12F&αGC (group 5 & 6) via intranasal instillation (*i.n.*, group 1–5) or subcutaneous injection (*s.c.*, group 6) for 3 times at 2 weeks intervals. Serum and saliva collected 2 weeks after the final vaccination were pooled-assessed via OPKA.
