# Supplementary material for: An intranasal cationic liposomal polysaccharide vaccine elicits humoral immune responses against Streptococcus pneumoniae
Source: Commun Biol. 2024 Sep 17;7:1158. doi: 10.1038/s42003-024-06806-1 (PMC11405767; doi:10.1038/s42003-024-06806-1)
Supplement: Supplementary file 4 — Reporting Summary [file 42003_2024_6806_MOESM4_ESM.pdf]

nature portfolio

Corresponding author(s): Mads H. Clausen

Last updated by author(s): Aug 5, 2024

# Reporting Summary

Nature Portfolio wishes to improve the reproducibility of the work that we publish. This form provides structure for consistency and transparency in reporting. For further information on Nature Portfolio policies, see our Editorial Policies and the Editorial Policy Checklist.

## Statistics

For all statistical analyses, confirm that the following items are present in the figure legend, table legend, main text, or Methods section.

| n/a | Confirmed | |
|---|---|---|
| ☐ | ☒ | The exact sample size (*n*) for each experimental group/condition, given as a discrete number and unit of measurement |
| ☐ | ☒ | A statement on whether measurements were taken from distinct samples or whether the same sample was measured repeatedly |
| ☐ | ☒ | The statistical test(s) used AND whether they are one- or two-sided<br>*Only common tests should be described solely by name; describe more complex techniques in the Methods section.* |
| ☐ | ☒ | A description of all covariates tested |
| ☐ | ☒ | A description of any assumptions or corrections, such as tests of normality and adjustment for multiple comparisons |
| ☐ | ☒ | A full description of the statistical parameters including central tendency (e.g. means) or other basic estimates (e.g. regression coefficient) AND variation (e.g. standard deviation) or associated estimates of uncertainty (e.g. confidence intervals) |
| ☐ | ☒ | For null hypothesis testing, the test statistic (e.g. *F*, *t*, *r*) with confidence intervals, effect sizes, degrees of freedom and *P* value noted<br>*Give P values as exact values whenever suitable.* |
| ☒ | ☐ | For Bayesian analysis, information on the choice of priors and Markov chain Monte Carlo settings |
| ☒ | ☐ | For hierarchical and complex designs, identification of the appropriate level for tests and full reporting of outcomes |
| ☒ | ☐ | Estimates of effect sizes (e.g. Cohen's *d*, Pearson's *r*), indicating how they were calculated |

*Our web collection on statistics for biologists contains articles on many of the points above.*

## Software and code

Policy information about availability of computer code

| | |
|---|---|
| Data collection | ASTRA 7.3.2 (AF4-MALS-dRI), Zetasizer 8.02 (DLS & zeta potential), BD FACSDiva 9.0.1 (CBA & Flow Cytometry), Leica Application Suite 4 (IHC), SparkControl 3.1 (ELISA) |
| Data analysis | FlowJo 10.8.2, Nist's Integrated Colony Enumerator (NICE), GraphPad Prism 9.5.0 |

For manuscripts utilizing custom algorithms or software that are central to the research but not yet described in published literature, software must be made available to editors and reviewers. We strongly encourage code deposition in a community repository (e.g. GitHub). See the Nature Portfolio guidelines for submitting code & software for further information.

## Data

Policy information about availability of data

All manuscripts must include a data availability statement. This statement should provide the following information, where applicable:

- Accession codes, unique identifiers, or web links for publicly available datasets
- A description of any restrictions on data availability
- For clinical datasets or third party data, please ensure that the statement adheres to our policy

Data will be made available on request.

## Research involving human participants, their data, or biological material

Policy information about studies with human participants or human data. See also policy information about sex, gender (identity/presentation), and sexual orientation and race, ethnicity and racism.

| | |
|---|---|
| Reporting on sex and gender | N/A |
| Reporting on race, ethnicity, or other socially relevant groupings | N/A |
| Population characteristics | N/A |
| Recruitment | N/A |
| Ethics oversight | N/A |

Note that full information on the approval of the study protocol must also be provided in the manuscript.

# Field-specific reporting

Please select the one below that is the best fit for your research. If you are not sure, read the appropriate sections before making your selection.

☒ Life sciences ☐ Behavioural & social sciences ☐ Ecological, evolutionary & environmental sciences

For a reference copy of the document with all sections, see nature.com/documents/nr-reporting-summary-flat.pdf

# Life sciences study design

All studies must disclose on these points even when the disclosure is negative.

| | |
|---|---|
| Sample size | Required sample sizes were estimated a priori with experiments believed to have similar estimated effect sizes. |
| Data exclusions | No data was excluded. |
| Replication | The N in each figure represents the number of independent replicates in each experiment. |
| Randomization | Mice were tagged, weighted, and then randomized in the software JMP with block size 8. |
| Blinding | Mice operation and data analysis were performed by different individuals. |

# Reporting for specific materials, systems and methods

We require information from authors about some types of materials, experimental systems and methods used in many studies. Here, indicate whether each material, system or method listed is relevant to your study. If you are not sure if a list item applies to your research, read the appropriate section before selecting a response.

## Materials & experimental systems

| n/a | Involved in the study | |
|---|---|---|
| ☐ | ☒ | Antibodies |
| ☐ | ☒ | Eukaryotic cell lines |
| ☒ | ☐ | Palaeontology and archaeology |
| ☐ | ☒ | Animals and other organisms |
| ☒ | ☐ | Clinical data |
| ☒ | ☐ | Dual use research of concern |
| ☒ | ☐ | Plants |

## Methods

| n/a | Involved in the study | |
|---|---|---|
| ☒ | ☐ | ChIP-seq |
| ☐ | ☒ | Flow cytometry |
| ☒ | ☐ | MRI-based neuroimaging |

## Antibodies

| | |
|---|---|
| Antibodies used | Fc block (BD Biosciences™ 553141), fixable viability stain 520 (BD Biosciences™ 564407), mouse PE-conjugated αGC-loaded mCD1d tetramer (Tetramer Shop MCD1d-001), hamster/rat anti-mouse CD3ε-BV605, CD69-APC, CD25-BV480, CD45R-APC-Cy7, CD27-BB700, CD138-BV421, and CXCR5-BV711 (BD Biosciences™ 563004, 560689, 566202, 561102, 742135, 562610, & BioLegend® 145529) |

| | |
|---|---|
| | biotinylated peanut agglutinin (PNA, VectorLabs B-1075-5)<br><br>HRP-conjugated goat anti-mouse IgM (abcam ab97230), IgA (abcam ab97235), IgGpoly (abcam ab6789), IgG3 (abcam ab97260), IgG1 (abcam ab97240), IgG2b (abcam ab97250), IgG2a (abcam ab97255) |
| Validation | Antibodies used were all validated by pretests in our lab. |

## Eukaryotic cell lines

Policy information about cell lines and Sex and Gender in Research

| | |
|---|---|
| Cell line source(s) | HL-60 cells were purchased from ATCC. |
| Authentication | The cell line were ordered directly from ATCC. No further authentication were performed. |
| Mycoplasma contamination | PCR test proved its not Mycoplasma contaminated. |
| Commonly misidentified lines<br>(See ICLAC register) | N/A |

## Animals and other research organisms

Policy information about studies involving animals; ARRIVE guidelines recommended for reporting animal research, and Sex and Gender in Research

| | |
|---|---|
| Laboratory animals | BALB/cJRj female mice from JANVIER LABS® were used for all experiments. |
| Wild animals | N/A |
| Reporting on sex | Only female were used in the study. We did not analyze the effect of biological sex as it was not the core point of our study. We want to protect the animal welfare. |
| Field-collected samples | N/A |
| Ethics oversight | Danish Animal Experiments Inspectorate |

Note that full information on the approval of the study protocol must also be provided in the manuscript.

## Plants

| | |
|---|---|
| Seed stocks | N/A |
| Novel plant genotypes | N/A |
| Authentication | N/A |

## Flow Cytometry

### Plots

Confirm that:

- [x] The axis labels state the marker and fluorochrome used (e.g. CD4-FITC).
- [x] The axis scales are clearly visible. Include numbers along axes only for bottom left plot of group (a 'group' is an analysis of identical markers).
- [ ] All plots are contour plots with outliers or pseudocolor plots.
- [ ] A numerical value for number of cells or percentage (with statistics) is provided.

### Methodology

| | |
|---|---|
| Sample preparation | Described in methods section "Cell phenotyping" |

| | |
|---|---|
| Instrument | BD© LSRFortessa™ |
| Software | BD FACSDiva 9.0.1 , FlowJo 10.8.2, , GraphPad Prism 9.5.0 |
| Cell population abundance | For each experiment, at least 100,000 events were acquired and analyzed. The viability ratio were all above 90%. |
| Gating strategy | iNKT cells (CD3ε+CD1d tetra:αGC+)<br>activated iNKT cells (CD3ε+CD1d tetra:αGC+CD69/25+)<br>follicular helper iNKT cells (CD3ε+CD1d tetra:αGC+CXCR5+)<br>plasmablast and plasma cells (CD27+CD138+) |

☒ Tick this box to confirm that a figure exemplifying the gating strategy is provided in the Supplementary Information.
